# Impact of household characteristics on patient outcomes post hip fracture: a Welsh nationwide observational cohort study

**DOI:** 10.1186/s12889-024-20766-5

**Published:** 2024-11-30

**Authors:** Clare MacRae, Anna Meyer, Stewart W. Mercer, Nazir Lone, Chris Dibben, Andrew D. Duckworth, Karin Modig, Bruce Guthrie

**Affiliations:** 1 Advanced Care Research Centre, University of Edinburgh, Bio Cube 1, Edinburgh BioQuarter, 13 Little France Road, Edinburgh, EH16 4UX UK; 2 Usher Institute, College of Medicine and Veterinary Medicine, University of Edinburgh, Edinburgh, UK; 3 Unit of Epidemiology, Institute of Environmental Medicine, Karolinska Institutet, Stockholm, Sweden; 4 Institute of Geography Edinburgh, University of Edinburgh Institute of Geography, Edinburgh, UK; 5 Edinburgh Orthopaedics, Royal Infirmary of Edinburgh, 51 Little France Crescent, Edinburgh, Midlothian EH16 4SA UK

**Keywords:** Hip fracture, Residence, Household, Multimorbidity, Care home admission, Health services research, Epidemiology, Older people

## Abstract

**Background:**

Hip fracture is common in older people and has significant health and care implications. This study aimed to examine the impact of household characteristics (living alone or living with someone who is themselves ill) on adverse outcomes following hip fracture.

**Methods:**

A cohort study of hip fracture patients aged ≥ 50 years living alone or with one co-resident using Welsh nationwide data between January 2013 and December 2018. Outcomes were emergency hospital admission within 30 days and care-home admission and mortality within one year of hospital discharge. Analysis used cause-specific Cox proportional hazards models to examine associations with living alone and with co-resident chronic disease status.

**Results:**

Of the 12,089 hip fracture patients discharged, 56.0% lived alone. Compared to hip fracture patients living with a co-resident, those living alone were more commonly women (78.4% versus 65.2%), older (mean 83.1 versus 78.5 years), and had more long-term conditions (mean 5.7 versus 5.3). In unadjusted analyses, compared to living with a co-resident with 0–1 long-term condition and no dementia, living alone (hazard ratio [HR] 1.44, 95%CI 1.23–1.68), living with someone with dementia (HR 1.57, 95%CI 1.07–2.30), and living with someone with 4 + physical long-term conditions (HR 1.24, 95%CI 1.03–1.49) were associated with an increase in mortality, but no significant association was found in adjusted analysis. Adjusted for age, sex, socioeconomic position, and long-term condition count of the hip fracture patient, living alone (adjusted HR [aHR] 2.26, 95%CI 1.81–2.81) and living with a co-resident with dementia (aHR 2.38, 95%CI 1.59–3.57) were both associated with more than double the risk of care home admission. There were no significant associations with 30-day hospital admission.

**Conclusions:**

Hip fracture patients who live alone have higher one-year mortality, but associations are explained by the demographic and clinical characteristics of those living alone. However, living alone or living with a co-resident with dementia was independently associated with an additional doubling of the risk of care home admission. Household-based approaches to research and health policy may help target risk groups following hip fracture community discharge and further research is needed to understand the mechanisms by which these associations act.

**Supplementary Information:**

The online version contains supplementary material available at 10.1186/s12889-024-20766-5.

## Background

Hip fracture is a serious injury associated with substantial healthcare costs [1, 2] that affects approximately 14.2 million people globally every year [3]. People with hip fractures are at increased risk of disability, impaired quality of life, chronic disease [3], and mortality [3, 4], as well as an increased likelihood of requiring long-term care [4].

Recovery from hip fracture is associated with restrictions in everyday life that include reduced mobility [5] and limitations on self-care and daily activities [6]. Health and care outcomes for individuals are known to be mediated by pre- [7] and intra-operative [8] characteristics of the individual. A small number of studies have examined how social support and cohabitation influence recovery following hip fracture, but the mechanisms of these associations are not well understood [9]. Existing evidence supports that living alone following a hip fracture [10], or experiencing infrequent social contact assessed as the number of social contacts immediately before a hip fracture is sustained [11], are both associated with a higher risk of higher mortality. Household co-residents – most often spouses and partners – are an important potential source of support to an older person with a hip fracture, but the effect of factors such as the chronic disease status of the co-resident remains insufficiently explored [12]. Older partners might have functional limitations themselves, thus having limited capacities to support their co-resident who has sustained a hip fracture. Additionally, living with a co-resident who has greater limitations such as dementia-related cognitive impairment might represent a risk factor for the hip fracture patient.

The extent to which the household unit can be leveraged as a point of intervention depends on whether cohabitation and co-resident chronic disease do indeed affect outcomes for hip fracture survivors. Therefore, the overarching aim of this study was to describe and classify households by characteristics likely to be important for care and recovery into which hip fracture patients are discharged by examining whether household factors relate to the likelihood of emergency hospital and care home admission and mortality.

## Methods

### Study design and cohort definition

Participants were people aged 50 years and above, living in Wales and registered with a general practitioner (GP) contributing data to the Secure Anonymised Information Linkage (SAIL) Databank. They had sustained a hip fracture between 01 January 2013 and 31 December 2018 and had no previous hip fracture in the previous year. We included participants who were discharged from hospital to households where they lived alone or with one co-resident. Patients who had died before discharge or were discharged from hospital directly to a care home were excluded. The SAIL Databank includes data for individuals registered with 80% of GP practices and 83% of all Welsh residents [13], therefore providing a comprehensive coverage of the population.

### Data sources

Hip fracture was defined as the presence of at least one hospital admission, identified in the Patient Episode Database for Wales (PEDW), with one of three International Classification of Diseases 10^th^ Revision codes at hospital discharge associated with hip fracture (S72.0 Fracture neck of femur, S72.1 Pertrochanteric fracture, S72.2 Subtrochanteric fracture) [14]. Demographic data including age, sex, and area socioeconomic position (SEP) quintile for the Welsh population (according to the Welsh Index of Multiple Deprivation [WIMD], which is a relative measure according to small geographical local area [15]) were derived from the Welsh Demographic Service Dataset (WDSD).

Chronic disease counts for both hip fracture survivors, and their co-residents where appropriate, were calculated on the date of hospital discharge of the hip fracture patient. Long-term conditions were defined using Read version 2 codes, prescribing, and laboratory data from the Welsh Longitudinal General Practice Dataset (WLGP) and ICD-10 codes from PEDW. The count of chronic disease was based on 47 long-term conditions, eight of which were mental health conditions, derived from the results of a recent Delphi consensus study recommending those to include in measurements of multimorbidity [16] (Supplementary Table 1). Methods used to ascertain long-term conditions were replicated from a recent study [17] (Supplementary Table 2) and included rules to ascertain active long-term conditions, such as asthma or depression, where a combination of recent prescribing and clinical coding was present to ensure that the long-term conditions were active at the time under evaluation in the study. Where measured, multimorbidity was defined as the presence of two or more long-term conditions [18].

Hip fracture patients were categorised into five mutually exclusive groups according to cohabitation and co-resident chronic disease status to allow direct comparison within statistical models. First, co-resident chronic disease was categorised into 0–1 long-term conditions (i.e., no multimorbidity), and then those with multimorbidity – according to the most commonly used definition of ≥ 2 long-term conditions [19] —were further categorised as 2–3 long-term conditions and ≥ 4 long-term conditions to examine whether there was a dose–response relationship between the number of co-resident long-term conditions and the examined outcomes for the study individuals. Second, we chose to incorporate the effect of dementia specifically, given that living with a person with dementia is associated with limited self-care, social isolation, and reduced emotional health [20]. Finally, given that living alone is associated with unplanned hospitalisation in the general population we included this as a distinct category within the exposure variable [21]. We, therefore, categorised living arrangements as follows: 1. living with one co-resident with zero or one long-term condition and no dementia (the reference category); 2. living with one co-resident with two or three long-term conditions and no dementia; 3. living with one co-resident with four or more long-term conditions and no dementia; 4. living with one co-resident with dementia, or 5. living alone.

### Study outcomes

The three study outcomes were emergency hospital (re)admission within 30 days of discharge from the index hip fracture admission (identified using hospital episode spell dates available in PEDW); admission to care home within the subsequent one-year (identified by linkage of WDSD with the RALF); and mortality incidence within the subsequent one-year (defined using registered deaths in the Annual District Death Extract [ADDE]). Identification of household co-residents was achieved through the linkage of the Anonymised Linkage Field (ALF) for individuals and the GP-registered addresses using the Residential Anonymised Linkage Field (RALF). The ALF and RALF fields are derived when identifiable data are sent to a trusted third party (TTP) within the Digital Health and Care Wales (DHCW). The TTP uniquely matches identities based on name, NHS number, date of birth, and Unique Property Reference Number (UPRN), using the Matching Algorithm for Consistent Results in Anonymised Linkage, which has an accuracy of 99.8% [22].

### Statistical analyses

Summary statistics comparing characteristics of hip fracture patients who lived alone with those who lived with one co-resident, and hip fracture patients with their co-residents. Differences were compared using the chi-squared test for categorical variables, and analysis of variance (ANOVA) for the comparison of means.

Rates per 1000 person-years with exact Poisson confidence intervals were calculated for each outcome. Associations between household exposure variables and time to event for mortality were examined using Cox proportional hazards (Cox PH) models. Fine and Grey competing risks survival analysis models were reported for non-mortality outcomes (emergency hospital admission and care home admission) to account for competing mortality (i.e., where an individual died and therefore could not have been admitted to a hospital or a care home beyond that time). While the Cox PH model analyses the risk of an event occurring, the Fine and Grey model specifically addresses competing risk of mortality by using the sub-distribution of the hazard to account for the competing risk, mortality in this case, and how this will alter the likelihood of the measured outcome from occurring. Both the Cox PH and Fine and Grey models assume proportionality of the hazards. This assumption was checked for each variable separately and no violations were found using cox.zph() function from the survival [23] package, and by visual inspection for deviation from the zero slope of plotted Schoenfeld residuals. Strength of associations of each covariate (including age as a continuous variable and an age quadratic term, sex, socioeconomic position, and number long-term conditions of the hip fracture survivor) in a univariate model and models with stepwise inclusion of covariates using the Akaike information criterion (AIC) as a measure of model fit. Data cleaning was performed using SQL to query IBM DB2 databases. Analyses were performed using the biostat, survival, and finalfit, and data visualisation using ggplot2 packages, performed using R version 4.1.2 [24].

The project received ethical approval from the SAIL Databank independent information governance panel [25]. The study was reported according to REporting of studies Conducted using Observational Routinely-collected Data reporting guidelines [26].

## Results

**Fig. 1:**
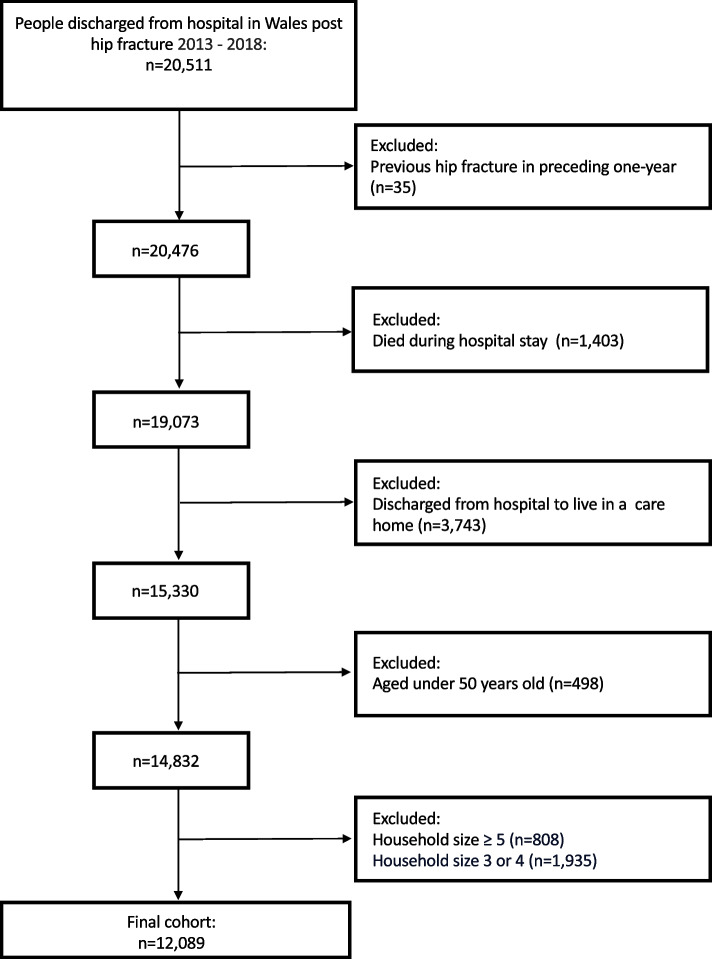
Study cohort selection flowchart

A total of 12,089 hip fracture patients were included in the study (Fig. 1). Characteristics of the study population are presented in Table 1: the majority of hip fracture patients were women (72.6%), with a mean age of 81.1 years, and a mean of 5.5 long-term conditions. A small proportion of hip fracture patients had zero (2.2%), one (5.0%), two (8.4%), or three (11.5%) long-term conditions, and the majority (72.8%) had four or more long-term conditions. A similar proportion of hip fracture patients lived in areas with the least deprived (19.3%) and most deprived (19.6%) SEP (Table 1).

**Table 1:** Characteristics of hip fracture survivors: total and stratified by cohabitation status

Characteristics n (%)	All hip fracture patients *N* = 12,089	Hip fracture patients living alone *N* = 6,771 (56.0%)	Hip fracture patients living with one co-resident *N* = 5,318 (44.0%)	p*
**Sex**				
**Women**	8775 (72.6)	5308 (78.4)	3467 (65.2)	< 0.001
**Age, years**				
**Mean (SD)**	81.1 (9.5)	83.1 (9.4)	78.5 (9.6)	< 0.001
**50–64**	749 (6.2)	316 (4.67)	433 (8.14)	< 0.001
**65–79**	3753 (31.0)	1579 (23.3)	2174 (40.9)	< 0.001
**80 +**	7587 (62.7)	4876 (72.0)	2711 (51.0)	< 0.001
**Number of long-term conditions**				
**Mean (SD)**	5.5 (3.0)	5.7 (2.9)	5.3 (3.1)	< 0.001
**0**	269 (2.2)	94 (1.4)	175 (3.3)	< 0.001
**1**	608 (5.0)	274 (4.0)	334 (6.3)	< 0.001
**2**	1020 (8.4)	509 (7.5)	511 (9.6)	< 0.001
**3**	1387 (11.5)	756 (11.2)	631 (11.9)	< 0.001
**4 +**	8805 (72.8)	5138 (75.9)	3667 (69.0)	< 0.001
**Socioeconomic position, quintiles**				
**5 (least deprived)**	2332 (19.3)	1181 (17.4)	1151 (21.6)	< 0.001
**4**	2341 (19.4)	1260 (18.6)	1081 (20.3)	< 0.001
**3**	2503 (20.7)	1384 (20.4)	1119 (21.0)	< 0.001
**2**	2539 (21.0)	1531 (22.6)	1008 (18.9)	< 0.001
**1 (most deprived)**	2374 (19.6)	1415 (20.9)	959 (18.0)	< 0.001

^*^Obtained from chi-squared or analysis of variance (ANOVA) as appropriate, test between living alone and living with one co-resident

Living alone was more common (56.0%) than living with one co-resident (44.0%). The hip fracture patients who lived alone were a higher proportion of women (5308/6771 [78.4%] versus 3467/5318 [65.2%]), were older (83.1 versus 78.5 years), had more long-term conditions (mean 5.7 versus 5.3), and more commonly lived in areas with highest deprivation (21.6% versus 17.4%) than those living with one co-resident (Table 1). Women were more likely to live alone (60.5%) than men (44.1%) (Supplementary Table 3).

The hip fracture patients who lived with one co-resident differed from their co-residents in several respects. Hip fracture patients were older (78.5 versus 71.3 years) and had a higher average number of long-term conditions (mean 5.3 versus 3.3 long-term conditions) than their co-residents. Co-residents most commonly had four or more long-term conditions and no dementia (37.1%); followed by 31.6% who had zero or one long-term condition and no dementia, and 28.0% with two or three long-term conditions and no dementia. The least common scenario was living with a co-resident with dementia, which was observed in only 3.4% of cases (Table 2).

**Table 2:** Hip fracture patients living with one co-resident: comparison between hip fracture patients and their co-residents

**Characteristic** **n (%)**	**Hip fracture patients living with one co-resident** ***N* = 5,318**	**Co-residents living with hip fracture patient** ***N* = 5,318**	***p***†
**Age (years)**			
**Mean (SD)**	78.5 (9.2)	71.3 (15.1)	< 0.001
**0 – 49**^a^	*NA*	499 (9.4)	< 0.001
**50—64**	433 (8.1)	922 (17.3)	< 0.001
**65—79**	2174 (40.9)	2035 (38.3)	0.032
**80 +**	2711 (51.0)	1862 (35.0)	< 0.001
**Number of long-term conditions**			
**Mean (SD)**	5.3 (3.1)	3.3 (2.8)	< 0.001
**0**	175 (3.3)	916 (17.2)	< 0.001
**1**	334 (6.3)	768 (14.4)	< 0.001
**2**	511 (9.6)	802 (15.1)	< 0.001
**3**	631 (11.9)	712 (13.4)	0.027
**4 +**	3667 (69.0)	2120 (39.9)	< 0.001
**Cohabitation and co-resident chronic disease**			
**Co-resident: 0–1 long-term conditions & no dementia**	1678 (31.6)	*NA*	*NA*
**Co-resident: 2–3 long-term conditions & no dementia**	1487 (28.0)	*NA*	*NA*
**Co-resident: 4 + long-term conditions & no dementia**	1971 (37.1)	*NA*	*NA*
**Co-resident: dementia**	182 (3.4)	*NA*	*NA*

^†^Obtained from chi-squared or analysis of variance (ANOVA) as appropriate, test between hip fracture patients and co-residents

^a^Hip fracture patients included in the study were aged 50 years and older, co-residents in the study we of any age

In the whole population, 343 (11.1%) hip fracture patients experienced emergency hospital admission within 30 days of hospital discharge, 1,514 (12.5%) were admitted to a care home and 1,751 (14.5%) died within one year of hospital discharge (Table 3). There were no statistically significant associations between cohabitation and co-resident chronic disease and the 30-day emergency hospital admission outcome in any of the models.

**Table 3:** Rate per 1000 person-years and hazard ratios (HRs) for mortality, emergency hospital admission, and care home admission by cohabitation and co-resident chronic disease

**Outcome**	**Exposure**	**Crude rate** **n/N**	**Rate per 1000 person-years** (95% CI^a^)	**Model 1** **Unadjusted HR** **(95%CI)**	**Model 2** **Partially adjusted HR**^b^ **(95%CI)**	**Model 3** **Fully adjusted HR**^c^ **(95%CI)**
**30-day emergency hospital admission**	Coresident: 0–1 long-term conditions & no dementia	176/1970	1364.7 (1170.5–1581.9)	*Reference*	*Reference*	*Reference*
	Coresident: 2–3 long-term conditions & no dementia	163/1509	1429.5 (1218.5–1666.6)	1.05 (0.85–1.30)	1.02 (0.83–1.27)	0.97 (0.78–1.20)
	Coresident: 4 + long-term conditions & no dementia	203/1657	1337.6 (1159.9–1534.8)	1.24 (1.03–1.49)	0.92 (0.75–1.12)	0.84 (0.69–1.03)
	Coresident: dementia	20/182	1450.8 (886.2–2240.7)	1.06 (0.67–1.69)	0.97 (0.61–1.54)	0.87 (0.54–1.38)
	Lives alone	781/6771	1507.5 (1403.7–1617.1)	1.10 (0.94–1.30)	1.02 (0.86–1.20)	0.96 (0.81–1.14)
**One-year care home admission**	Coresident: 0–1 long-term conditions & no dementia	89/1970	55.5 (44.6–68.3)	*Reference*	*Reference*	*Reference*
	Coresident: 2–3 long-term conditions & no dementia	111/1509	79.2 (65.1–95.4)	1.42 (1.07–1.88)	1.38 (1.04–1.83)	1.32 (1.00–1.74)
	Coresident: 4 + long-term conditions & no dementia	153/1657	82.4 (69.9–96.6)	1.47 (1.14–1.91)	1.27 (0.98–1.65)	1.20 (0.93–1.56)
	Coresident: dementia	32/182	204.3 (139.7–288.4)	3.52 (2.35–5.27)	2.59 (1.73–3.88)	2.38 (1.59–3.57)
	Lives alone	1129/6771	193.2 (182.1–204.8)	3.34 (2.69–4.15)	2.33 (1.88–2.90)	2.26 (1.81–2.81)
**One-year mortality**	Coresident: 0–1 long-term conditions & no dementia	189/1970	120.5 (104.0–139.0)	*Reference*	*Reference*	*Reference*
	Coresident: 2–3 long-term conditions & no dementia	195/1509	141.8 (122.6–163.2)	1.18 (0.96–1.44)	1.13 (0.93–1.38)	1.06 (0.87–1.29)
	Coresident: 4 + long-term conditions & no dementia	271/1657	149.9 (132.5–168.8)	1.24 (1.03–1.49)	1.11 (0.92–1.33)	0.99 (0.82–1.20)
	Coresident: dementia	31/182	189.9 (129.0–269.6)	1.57 (1.07–2.30)	1.29 (0.88–1.88)	1.11 (0.76–1.62)
	Lives alone	1071/6771	174.0 (163.7–184.7)	1.44 (1.23–1.68)	1.19 (1.02–1.39)	1.12 (0.95–1.31)

^a^Poisson confidence intervals

^b^Adjusted for hip fracture patient characteristics: age (years) continuous, age (years) quadratic, sex, socioeconomic position (SEP)

^c^Adjusted for hip fracture patient characteristics: age (years) continuous, age (years) quadratic, sex, socioeconomic position (SEP), number of long-term conditions

People who lived alone had higher rates of admission to care homes in the subsequent year than people who lived with a co-resident with minimal chronic disease (0–1 long-term conditions and no dementia). This was found in both unadjusted analyses (rate per 1000 person-years 193.2 [95%CI 182.1–204.8] versus 55.5 [95%CI 44.6–68.3] respectively, hazard ratio [HR] 3.34 [95%CI 2.69–4.15]), and persisted when adjusted for age, sex, SEP, and number of long-term conditions of the hip fracture patient (Model 3) with more than double the risk (adjusted HR [aHR] 2.26 [95%CI 1.81–2.81]). Living with a co-resident with dementia compared to living with a co-resident with minimal chronic disease (0–1 long-term conditions and no dementia) was also associated with one-year care home admission in unadjusted analyses (rate per 1000 person-years 204.3 [95%CI 139.7–288.4] versus 55.5 [95%CI 44.6–68.3] and HR 3.52 [95%CI 2.35–5.27]), and this association was also persistent in the adjusted model (Model 3) (aHR 2.38 (1.59–3.57).

**Fig. 2:**
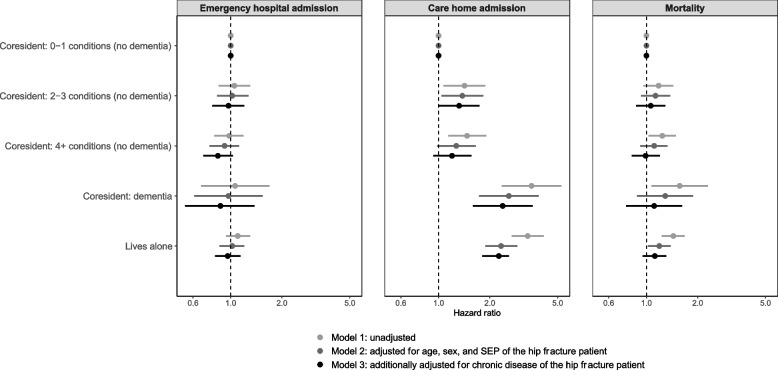
Hazard ratios for adverse outcomes by cohabitation and co-resident chronic disease. Model one was unadjusted, model two was adjusted for age, sex, and socioeconomic position (SEP) of the hip fracture patient, and model three was additionally adjusted for chronic disease (number of long-term conditions) of the hip fracture patient

Several associations were identified with one-year mortality. Hip fracture patients living with a co-resident with four or more long-term conditions and no dementia compared to living with a co-resident with minimal chronic disease (0–1 long-term conditions and no dementia) had higher unadjusted rates and associations with mortality (rate per 1000 person-years 149.9 [95%CI 132.5–168.8] versus 120.5 [95%CI 104–139], HR 1.24 [95%CI 1.03–1.49]) but this was attenuated when adjusted for age, sex, SEP, and number of long-term conditions of the hip fracture patient (Model 3) (aHR 0.99 [95%CI 0.82–1.20]). The association with one-year mortality following hospital discharge persisted for those who lived alone versus living with a co-resident with minimal chronic disease (0–1 long-term conditions and no dementia) after adjustment for age, sex, SEP of the hip fracture patient (Model 2) (HR 1.19, 95%CI 1.02–1.39), but was attenuated when the number of long-term conditions of the hip fracture patient (Model 3) was included in the adjustment (aHR 1.12 [95%CI 0.95–1.31]) (Fig. 2, Table 3, and Supplementary Table 4).

## Discussion

This study finds that living alone or living with a co-resident with dementia was independently associated with an additional doubling of the risk of care home admission. Hip fracture patients who lived alone or lived with someone with dementia had higher one-year mortality and care home admissions than those who lived with a co-resident minimal chronic disease in unadjusted analysis. There were no statistically significant associations between cohabitation and co-resident chronic disease and emergency hospital admission in any analysis, and the number of long-term conditions other than dementia that co-residents had was not associated with any outcome in the adjusted analysis.

The findings are consistent with Jorgensen et al., [27] who examined data for 35,066 hip fracture patients living in Sweden aged 65 years and over and found that more women (71.6%) than men (52.2%) with hip fractures lived alone. Living alone was not associated with hospital readmission, although it was associated with a higher probability of initiation of or increase in the number of hours of home care [27]. Our study found that associations with mortality were explained by the demographic and clinical characteristics of those living alone (as they are older and have more long-term conditions than people with hip fracture patients living with another person). These findings are consistent with research using the Norwegian NOREPOS Hip Fracture Database found that living alone versus living with a partner was associated with an increased risk of mortality in people with hip fracture in adjusted analysis (HR 1.28, 95%CI 1.16–1.43). However, adjustment did not include accounting for the number of long-term conditions of the hip fracture patient [28] and is therefore similar to the statistically significant association observed in our partially adjusted Model Two which did not persist in fully adjusted analysis.

Strengths of this study include examination of hip fracture patients surviving to hospital discharge from a large nationwide sample over a six-year period. Identification of household units and co-residents, and the chronic disease counts for both the hip fracture survivor and co-residents, were ascertained on the day of hospital discharge of each hip fracture patient. The analyses used long-term conditions recommended for use in multimorbidity research [16], incorporating multiple data sources (primary care and hospital inpatient condition coding, plus prescription and laboratory results) that have been shown to improve the representativeness of multimorbidity measured in the same population [17]. Survival modelling appropriately accounted for competing mortality risks, which is particularly important for the care home admission outcome since death is more common than care home admission over one year of follow-up.

Limitations include that information on functional status, cognitive function, and the provision of informal and formal in-home care was not available because the study used data from electronic health records (EHRs) which do not reliably record this information. Information regarding the severity of the co-resident long-term conditions was not incorporated into the analyses which also relates to the limitations of research using EHRs. Despite substantial research attention and the use of innovative approaches, there has been a failure to achieve effective ascertainment of long-term conditions severity in EHRs [29] and markers of disease severity in EHRs have not been found to contribute meaningfully to the prediction of activities of daily living, mortality, or other health outcomes [30]. Analysis was exploratory, and like all observational analyses, it is not possible to be certain that observed associations are causal, but the observed associations with care home admission are relatively large. Due to the small number of hip fracture patients admitted to care homes, it was not possible to examine different routes of admission to care home, for example comparing those admitted to a care home directly from home or following an emergency hospital admission. The effect of household composition on the measured outcomes might vary between the different age groups within the study, however, even when using a whole population sample as in this study, stratification by age group resulted in small numbers precluding interpretable results when taking this approach. Finally, the analysis was only of people with hip fracture living alone or with one other person because accounting for co-resident status in larger households is challenging (although 82% of people with hip fracture live in one or two-person households).

Our study found that people who lived alone or with a co-resident with dementia had a higher risk of admission to a care home. One possible explanation is that the potential to receive care within the household is a major factor in determining the likelihood of care home admission. A study by McCann et al [31] used census data for 2,138 adults from a sample of older adults living in Northern Ireland and found that women living alone, compared to those living with a partner, were at a substantially increased risk of care home admission (unadjusted HR 1.81 {95%CI 1.17–2.81], adjusted for age and health status HR 1.74 [95%CI 1.12–2.70]). They proposed that this association was partly explained by the health characteristics of people who lived alone, who were older and had more long-term conditions, but that the remaining independent association was likely to relate to informal domiciliary support and social isolation. Another potential explanation is that living alone is associated with loneliness. The authors Hanratty et al., [32] using a sample of adults in the English Longitudinal Study of Ageing (ELSA), found that loneliness was associated with an independently increased risk of admission to a care home. Although both of these studies included samples of older adults from the general population, rather than those with hip fracture, the mechanisms are likely to be similarly relevant, or even exaggerated, in those with hip fracture given the functional limitations and increased care needs common to those affected. The authors of the NOREPOS study [28] hypothesise that the consequences of hip fracture for people who live alone might be more marked than for those who cohabit with others, leading to those living alone being at greater risk of mortality. Mechanisms of the increased risk of adverse outcomes experienced by people who live alone remain relatively unexplored and are likely to be related to receiving less social support than those who co-habit [11] but might be explained by other mechanisms such as elevated inflammatory responses found in people experiencing social isolation that is thought to induce detrimental health consequences [23]. A study of mortality risk in participants of the English Longitudinal Study of Ageing (not specific to people with hip fracture), similarly found a relationship between living alone with all-cause mortality, that the authors interpreted as relating at least in part to loneliness and/or depression [33]. However, associations with loneliness and depression are likely to be complicated by the reasons why someone lives alone, for example, separation, divorce, and bereavement [33], and further understanding of these mechanisms is needed. There is a need for replication of this study in a larger dataset with the availability of similar data regarding household living arrangements and health and care outcomes, but the findings are consistent with previous research that household context matters. Future research is also needed to further examine the impact of household characteristics in different datasets and other clinical populations, including the development of methods to classify households with greater numbers of residents.

## Conclusions

In conclusion, this study finds that the large minority of hip fracture patients who live alone were older and have more chronic diseases than those who live with someone else. They had an increased risk of one-year mortality (explained by their individual characteristics) and care home admission (in addition to the risk associated with their individual characteristics). A similar risk of care home admission was observed in people with hip fracture living with someone with dementia, although this was uncommon compared to living alone. Research to better understand the importance and mechanisms by which household context is associated with outcomes is needed.

## Supplementary Information

Supplementary Material 1.

## Data Availability

No datasets were generated or analysed during the current study.
