# Supplementary material for: Impact of household characteristics on patient outcomes post hip fracture: a Welsh nationwide observational cohort study
Source: BMC Public Health. 2024 Nov 30;24:3344. doi: 10.1186/s12889-024-20766-5 (PMC11607986; doi:10.1186/s12889-024-20766-5)
Supplement: Supplementary file 1 — Supplementary Material 1. [file 12889_2024_20766_MOESM1_ESM.docx]

**Supplementary File.**

# Supplementary Table 1. Choice of long-term conditions in relation to those included by “Measuring multimorbidity in research: a Delphi consensus study” (Ho et al, 2022)^16^

| **Body system as defined by Delphi study** | **Delphi “always include” long-term conditions** | **Delphi “usually include” long-term conditions** | **Inclusion (including aggregation into higher-level long-term conditions) or exclusion (and rationale)** |
| --- | --- | --- | --- |
| Cardiovascular system | Stroke | - | Included (as stroke AND transient ischaemic attack combined) |
|  | Coronary artery disease | - | Included |
|  | Heart failure | - | Included |
|  | Peripheral arterial disease | - | Included |
|  | - | Heart valve disorders | Included |
|  | - | Arrhythmia | Included |
|  | - | Venous thromboembolic disease | Included |
|  | - | Aneurysm | Included |
|  |  | Hypertension | Included |
| Metabolic and endocrine disease | Diabetes | - | Included |
|  | Addison's disease | - | Included |
|  | Cystic fibrosis | - | Included |
|  | - | Thyroid disorders | Included |
| Respiratory disease | Chronic obstructive pulmonary disease | - | Included |
|  | Asthma | - | Included |
|  | - | Bronchiectasis |  |
| Neurological disease | Parkinson's disease | - | Included |
|  | Epilepsy | - | Included |
|  | Multiple sclerosis | - | Included |
|  | Paralysis | - | Included |
|  |  | Transient ischaemic attack | Included (as stroke AND transient ischaemic attack combined) |
|  | - | Peripheral neuropathy | Included |
|  | - | Chronic primary pain | Excluded: it was decided to exclude based on CPRD @ Cambridge prescribing code list (<https://www.phpc.cam.ac.uk/pcu/research/research-groups/crmh/cprd_cam/codelists/v11/>) where listed analgesics could result in double counting of long-term conditions. It was deemed difficult to avoid with the use of a broad range of analgesic medications used for mild to moderate pain, for example, osteoarthritis for paracetamol, codeine-containing compounds, or non-steroidal anti-inflammatory drugs, and cancer for strong analgesics such as morphine |
| Cancer | Solid organ cancers | - | Included (as cancer) |
|  | Haematological cancers (included as cancer) | - | Included (as cancer) |
|  | Metastatic cancers (included as cancer) | - | Included (as cancer) |
|  | - | Melanoma (included as cancer) | Included (as cancer) |
|  | - | Cerebral tumours that can cause disability | Excluded: it was decided to exclude based on the difficulty in defining this population using code lists applied to routinely collected data |
| Mental and behavioural disorder | Dementia | - | Included |
|  | Schizophrenia | - | Included |
|  |  | Depression | Included |
|  |  | Bipolar disorder | Included |
|  |  | Drug or alcohol misuse | Included |
|  |  | Eating disorder | Included |
|  |  | Autism | Included |
|  |  | Post-traumatic stress disorder | Included |
| Musculoskeletal disease | Connective tissue disease |  | Included |
|  |  | Osteoarthritis | Included |
|  | - | Long-term musculoskeletal problems due to injury | Excluded: our group decided to exclude due to difficulty in accurately coding and identifying which long-term conditions cause long-term impairment |
|  | - | Osteoporosis | Included |
|  |  | Gout | Included |
| Urogenital disorder | Chronic kidney disease |  | Included |
|  | End-stage kidney disease (included as chronic kidney disease) | - | Included |
|  | - | Endometriosis | Included |
|  |  | Chronic urinary tract infection | Excluded: it was decided to exclude due to difficulty in accurately coding and identifying which long-term conditions cause long-term impairment. Using antimicrobial prescribing other than trimethoprim and nitrofurantoin also presents difficulties because these agents are not specific to infections of the urinary tract and broader spectrum antimicrobial agents could be used to treat acute soft tissue and respiratory infections |
| Haematological disorder | - | Anaemia | Included |
| Eye disease | - | Vision impairment that cannot be corrected | Included |
| Ear disease | - | Hearing impairment that cannot be corrected | Included |
|  | - | Meniere's disease | Included |
| Infectious disease | HIV | - | Excluded: not accessible in SAIL Databank |
|  |  | Chronic Lyme disease | Excluded: it was decided to exclude due to difficulty in accurately coding and identifying which long-term conditions cause long-term impairment |
|  | - | Tuberculosis | Included |
|  |  | Post-acute covid-19 (study pre-2020) | Study period pre-2020 |
| Congenital disease | - | Congenital disease and chromosomal abnormalities | Included |
| Digestive disease | Chronic liver disease | - | Included |
|  | Inflammatory bowel disease | - | Included |
|  | - | Chronic pancreatitis | Included |
|  | - | Peptic ulcer | Included |

# Supplementary Table 2. List of included long-term conditions including explanation of rules and code-lists used to define long-term conditions.

| **Condition name** | **Implementation rules** | **Code list or code list Read v2***^,^** | **Code list or code list ICD-10***^,^** |
| --- | --- | --- | --- |
| Addison’s disease | Read v2 or ICD-10 code ever recorded | A176., C154., C1540, C1541, C1540, C154z | E271, E272, E271, E274 |
| Alcohol and substance misuse | Read v2 or ICD-10 code ever recorded | c945 - alcohol misuse,  c1594 - substance misuse | c947 - alcohol misuse,  1596 - substance misuse |
| Anaemia | Read v2 or ICD10 code 12-month look back (iron deficiency, B12 deficiency, folate deficiency), Read v 2 or ICD10 code ever recorded (aplastic anaemia, sickle cell anaemia, thalassaemia) | \| c831 – iron deficiency anaemia, c979 - aplastic anaemia, c1013 - B12 deficiency anaemia, c1174 - folate deficiency anaemia, c1556 - sickle cell anaemia, c1603 - thalassaemia \| \| --- \| | c833 - iron deficiency anaemia, c981 - aplastic anaemia, c1015 - B12 deficiency anaemia, c1176 - folate deficiency anaemia, c1558 - sickle cell anaemia, c1602 - thalassaemia |
| Aneurysm | Read v2 or ICD-10 code ever recorded | c783 - abdominal aortic aneurysm | c785 - abdominal aortic aneurysm |
| Anxiety | Read v2 or ICD-10 code in the previous 12 months OR 4 or more anxiolytic/hypnotic prescriptions in the previous 12 months | c976 - anxiety | c978 - anxiety |
| Arrhythmia | Read v2 or ICD-10 code ever recorded | c789 - atrial fibrillation, c915 – supraventricular tachycardia, c1010 – atrioventricular block complete  c1553 - sick sinus syndrome, c1615 - trifasicular block | c791 - atrial fibrillation, c917 – supraventricular tachycardia, c1012 – atrioventricular block complete, c1555 - sick sinus syndrome, c1617 - trifasicular block |
| Asthma | Read v2 or ICD-10 code ever recorded AND any prescription in the last 12 months AND no Read v2 or ICD-10 Chronic Obstructive Pulmonary Disease code ever recorded | c2418 - asthma | c994 - asthma |
| Autism | Read v2 or ICD-10 code ever recorded | c995 - autism | c997 - autism |
| Bipolar affective disorder | Read v 2 or ICD-10 code ever coded OR lithium ever prescribed | c793 - bipolar | c795 - bipolar |
| Bronchiectasis | Read v2 or ICD-10 code ever recorded | c1045 - bronchiectasis | c1047 - bronchiectasis |
| Coronary artery disease | Any Read v2 or ICD-10 code ever recorded | c802 – coronary heart disease, c1296 – myocardial infarction, c1588 - stable angina, c1631 - unstable angina | \| c804 – coronary heart disease, c1298 – myocardial infarction, c1590 - stable angina, c1633 – unstable angina \| c1590 - stable angina \| c1633 - unstable angina \| \| --- \| --- \| --- \| |
| Cancer (Delphi: Solid organ cancers, haematological cancer, metastatic cancer, melanoma) | First Read v2 or ICD-10 code in the previous 12 months | c846 – myelodysplastic, c858 - non-Hodgkin’s lymphoma, c1218 - Hodgkin’s lymphoma, c1258 – leukaemia, c1361 - primary myeloma, c1415 - primary melanoma, c1385 - primary biliary, c1388 - primary bladder, c1391 - primary bone, c1397 - primary brain, c1400 - primary breast, c1403 - primary cervical, c1406 - primary kidney, c1409 - primary liver, c1412 - primary lung, c1418 - primary mesothelioma, c1421 - primary multiple, c1424 - primary oesophageal, c1430 - primary other, c1433 - primary ovarian, c1436 - primary pancreatic, c1439 - primary prostate, c1445 - primary stomach, c1448 - primary testicular, c1454 - primary thyroid, c1457 - primary uterine, c1513 - secondary lymph nodes, c1516 - secondary adrenal, c1519 - secondary bone, c1522 - secondary bowel, c2065 - bowel cancer primary or secondary, c1525 - secondary brain, c1528 - secondary liver, c1531 - secondary lung, c1540 - secondary peritoneum, c1543 - secondary pleura, c1537 - secondary other | c848 – myelodysplastic, c860 - non-Hodgkin’s lymphoma, c1220 - Hodgkin’s lymphoma, c1260 – leukaemia, c1363 - primary myeloma, c1417 - primary melanoma, c1387 - primary biliary, c1390 - primary bladder, c1393 - primary bone, c1399 - primary brain, c1402 - primary breast, c1405 - primary cervical, c1408 - primary kidney, c1411 - primary liver, c1414 - primary lung, c1420 - primary mesothelioma, c1423 - primary multiple, c1426 - primary oesophageal, c1432 - primary other, c1435 - primary ovarian, c1438 - primary pancreatic, c1441 - primary prostate, c1447 - primary stomach, c1450 - primary testicular, c1450 - primary thyroid, c1459 - primary uterine, c1515 - secondary lymph nodes, c1518 - secondary adrenal, c1521 - secondary bone, c1524 - secondary bowel, c1527 - secondary brain, c1530 - secondary liver, c1533 - secondary lung, c1542 - secondary peritoneum, c1545 - secondary pleura, c1539 – secondary other |
| Cystic fibrosis | Any Read v2 or ICD-10 or Read v2 code ever recorded  AND NO bronchiectasis Read v2 or ICD-10 code ever recorded | C799 – cystic fibrosis | c801 - cystic fibrosis |
| Chromosomal abnormalities† | Any Read v2 or ICD-10 code ever recorded | PJ00., PJ01., PJ01., PJ0.., PJ0.., PJ0z., PJ0z., PJ0.., PJ02., PJ02., PJ20., PJ21., PJ21., PJ2.., PJ2z., PJ2z., PJ22., PJ10., PJ11., PJ11., PJ1.., PKyz0, PJ1z., PJ1z., PJ12., PJ50w, PJ50x, PJ50x, PJ510, PJ511, PJ520, PJ521, PJ523, PJ524, PJ513, PJ513, PJ515, PJ515, PJ512, PJ503, PJ514, PJ514, PJ500, PJ501, PJ502, PJ503, PJ504, PJ505, PJ506, PJ0.., PJ508, PJ50., PJ51., PJ51z, PJz3., PJ50z, PyuA0, PJ507, PJ50y, PJ52., PJ52z, PJ36., PJ370, PJ37., PJ37z, PJ37., PJ38., PJ32., PJ31., PJ338, PJ339, PKyz7, PKyz5, PKyz7, PKyz5, PJ330, PJ30., PJz2., PJ3z., PJ30.11, PyuA1, PJ33., PJ33z, PJ3y., PJ331, PJ332, PJ331, PJ332, PJ332, PJ331, PJ333, PJ336, PJ337, PJ334, PJ34., PJ35., PJ3.., PyuA2, PJ37., PJ531, PJ632, PJ633. PJ60., PJy10, PJ635, PJ630, PJ636, PJ634, PJ63., PJ63z, PJ63z, PyuA5, PJ636, PJ63z, PJ6.., PJ6z., PJ64., PJ64z, PJ62., PJ631, PJy2., PJy2., PJy4., PJy5..PJ640, PyuA6, PJy12, PJyy0, PJyy1, PJyy4, PJyy2, F1y0., PyuAB, PJy1z, PJy1., PJy13, PJyy., PyuAD | Q968, Q969, Q97, Q970, Q971, Q972, Q973, Q978, Q979, Q980, Q99, Q990, Q991, Q992, Q998, Q999, Q87, Q870, Q871, Q872, Q873, Q874, Q875, Q878, Q878 |
| Chronic kidney disease | Any coding as per PC implementation rules OR ICD-10 code ever recorded | \| c811 - end-stage renal disease  c2035 - chronic kidney disease \| \| --- \| | \| c813 - end-stage renal disease  c2846 - chronic kidney disease \| \| --- \| |
| Chronic liver disease | Read v2 or ICD-10 code ever recorded | c998 - autoimmune liver disease, c1373 - portal HTN, c1082 - liver fibrosis, sclerosis, and cirrhosis, c1265 - alcoholic liver disease, c1645 - oesophageal varices, c1073 – chronic viral hepatitis | c1000 - autoimmune liver disease, c1375 - portal HTN, c1084 - liver fibrosis, sclerosis, and cirrhosis, c1267 - alcoholic liver disease, c1647 - oesophageal varices, c1075 chronic viral hepatitis |
| Chronic obstructive pulmonary disease | Read v2 or ICD-10 code ever recorded | c2748 – chronic obstructive pulmonary disease | c2746 - chronic obstructive pulmonary disease |
| Connective tissue disorders | Read v2 or ICD-10 code ever recorded | c815 – giant cell arteritis, c887 – polymyalgia rheumatica, c905 – rheumatoid arthritis,  c912 – systemic lupus erythematosus, c961 - ankylosing spondylosis,  c1249 - juvenile arthritis, c1565 – Sjogren syndrome, c890 - psoriatic arthritis | c817 – giant cell arteritis, c889 – polymyalgia rheumatica, c907 – rheumatoid arthritis,  c914 – systemic lupus erythematosus, c963 - ankylosing spondylosis, c1251 juvenile arthritis, c1567 Sjogren syndrome, c892 psoriatic arthritis |
| Dementia | Read v2 or ICD-10 code ever recorded | c2777 - dementia | c2773 - dementia |
| Depression | Read v2 or ICD-10 code in the previous 12 months OR 4 or more antidepressant prescriptions in the previous 12 months | c1111 - depression | c1113 - depression |
| Diabetes | Read v2 or ICD-10 code ever recorded | c1120 – diabetes, c1128 - diabetes neurological complications, c1117 - diabetes eye complications | c1122 – diabetes, c1130 - diabetes neurological complications, c1119 - diabetes eye complications |
| Eating disorder | Read v2 or ICD-10 code ever recorded | C3252 - eating disorder | c2935 - eating disorder |
| Endometriosis | Any Read v2 or ICD-10 code ever recorded AND age < 55 years | c1145 - endometriosis | c1147 - endometriosis |
| Epilepsy | Read v2 or ICD-10 code ever coded AND any antiepileptic (excluding gabapentinoids) prescription in the previous12 months | c1154 - epilepsy | c1156 - epilepsy |
| Gout | Read v2 or ICD-10 code ever recorded | c1191 - gout | c1193 - gout |
| Hearing impairment | Read v2 or ICD-10 code ever recorded | c1102 - hearing loss | c1104 - hearing loss |
| Heart failure | Read v2 or ICD-10 code ever recorded | c1206 - heart failure | c1208 - heart failure |
| Heart valve disorders | Read v2 or ICD-10 code ever recorded | c908 - rheumatic heart valve, c1289 - multiple heart valve disorder, c1308 - nonrheumatic aortic valve disorder, c1311 - nonrheumatic mitral valve disorder | c910 - rheumatic heart valve disorder, c1291 - multiple heart valve disorder, c1310 - nonrheumatic aortic valve disorder, c1313 - nonrheumatic mitral valve disorder |
| Hypertension | Read v2 or ICD-10 code ever recorded | c1227 - hypertension | c1229 - hypertension |
| Inflammatory bowel disease | Read v2 or ICD-10 code ever recorded | c1096 - Crohn's disease, c1621 - ulcerative colitis | c1098 - Crohn's disease, c1623 - ulcerative colitis |
| Meniere’s disease | Read v2 or ICD-10 code ever recorded | c1279 - Meniere's disease | c1281 - Meniere's disease |
| Multiple sclerosis | Read v2 or ICD-10 code ever recorded | c855 – Multiple Sclerosis | C857 – Multiple sclerosis |
| Osteoarthritis | Read v2 or ICD-10 code ever recorded | c861 - osteoarthritis | c863 - osteoarthritis |
| Osteoporosis | Read v2 or ICD-10 code ever recorded | c1326 - osteoporosis | c1328 - osteoporosis |
| Peripheral arterial disease | Read v2 or ICD-10 code ever recorded | c1349 – peripheral arterial disease | c1351 - peripheral arterial disease |
| Pancreatitis (chronic) | Read v2 or ICD-10 code ever recorded | 14CG., J671. | K861, K860 |
| Paralysis | Read v2 or ICD-10 code ever recorded | F2411, F141., F241., F2410, F2300, F240.. F2401, F2400, F232., F232.. F240., F242.. F230., F230z, F2301 | G82, G830, G808, G801, G803 |
| Parkinson’s disease | Read v2 or ICD-10 code ever recorded | c896 – Parkinson’s disease | c898 - Parkinson’s disease |
| Peptic ulcer | Read v2 or ICD-10 code ever recorded | c1624 - peptic ulcer | c1626 - peptic ulcer |
| Peripheral neuropathy | Read v2 or ICD-10 code ever recorded | c1346 - peripheral neuropathy | c1348 - peripheral neuropathy |
| Post-traumatic stress disorder | Read v2 or ICD-10 code ever recorded | E2831, E29y1, Eu431, Eu433, Eu434, ZS7C7 | F431 |
| Schizophrenia | Read v2 or ICD-10 code ever recorded | c1503 - schizophrenia | c1503 - schizophrenia |
| Stroke and transient ischaemic attack | Read v2 or ICD-10 code ever recorded | c834 - intracerebral haemorrhage, c837 - ischaemic stroke, c918 - NOS stroke, c921 - subarachnoid haemorrhage, c921, c927 – transient ischaemic attack | c836 - intracerebral haemorrhage, c839 - ischaemic stroke, c920 - NOS stroke, c923 - subarachnoid haemorrhage, c929 transient ischaemic attack |
| Tuberculosis | Read v2 of ICD-10 code in the previous 5 years | c924 - tuberculosis | c926 - tuberculosis |
| Thyroid disorders | Read v2 or ICD-10 code ever recorded | C1609 - thyroid | C1611 - thyroid |
| Visual impairment | Read v2 or ICD-10 code ever recorded | c1041 - visual impairment and blindness | c1043 - visual impairment and blindness |
| Venous thromboembolic disease | Read v2 or ICD-10 code in previous 12-months or >1 code ever | c880 – pulmonary embolism,  c1657 - deep vein thrombosis | c882 – pulmonary embolism,  c1659 – deep vein thrombosis |

NA = not applicable, long-term condition not included in Barnett et al^34^ analysis

*Read v2 codes truncated to 5 digits for compatibility with SAIL Databank.

**Code lists from the HDR UK Phenotype Library are formatted as cXXX (e.g., c882 is pulmonary embolism) where they are called ‘concepts’ and can be downloaded by searching for the code list/concept number at <https://phenotypes.healthdatagateway.org/concepts/>?

†Available at OpenSAFELY <https://github.com/opensafely/hdruk-os-covid-paeds/commit/6295c353125577798fafe9afa25d882a1b911200>

**Supplementary Table 3. Characteristics of hip fracture survivors: total and stratified by sex and co-resident status.**

Women who lived alone were older (84.0 versus 78.2 years), and on average had more long-term conditions (mean 5.6 versus 5.1) than those living with a co-resident. Similar but smaller differences were seen in men living alone, who were also older (80.0 versus 79.1 years) and had more long-term conditions (5.8 versus 5.7) than those living with one co-resident.

| **Characteristics** | **All hip fracture**  **survivors**  **N = 12,089** | **Lives alone** | | | | **Lives with co-resident** | | | |
| --- | --- | --- | --- | --- | --- | --- | --- | --- | --- |
|  |  | **Total**  **N = 3314** | **Men**  **N = 1463** | **Women**  **N = 1851** | **p*** | **Total**  **N = 8775** | **Men**  **N = 5308** | **Women**  **N = 3467** | **p*** |
| **Age, years** |  |  |  |  |  |  |  |  |  |
| **Mean (SD)** | 81.1 (9.5) | 83.1 (9.2) | 83.1 (9.2) | 84.0 (8.4) | <0.001 | 78.5 (9.2) | 79.1 (9.1) | 78.2 (9.3) | <0.001 |
| **50-64** | 749 (6.2) | 316 (4.7) | 316 (4.7) | 152 (2.9) | <0.001 | 433 (8.1) | 139 (7.5) | 294 (8.5) | 0.007 |
| **65-79** | 3753 (31.0) | 1579 (23.3) | 1579 (23.3) | 1161 (21.9) |  | 2174 (40.9) | 714 (38.6) | 1460 (42.1) | <0.001 |
| **80+** | 7587 (67.7) | 4876 (72.0) | 4876 (72.0) | 3995 (75.3) |  | 2711 (51.0) | 998 (53.9) | 1713 (49.4) | <0.001 |
| **Number of long-term conditions** |  |  |  |  |  |  |  |  |  |
| **Mean (SD)** | 4.6 (2.8) | 5.7 (2.9) | 5.8 (3.1) | 5.6 (2.9) | 0.0017 | 5.3 (3.1) | 5.7 (3.1) | 5.1 (3.1) | <0.001 |
| **0** | 494 (4.1) | 94 (1.4) | 19 (1.3) | 75 (1.4) | 0.905 | 175 (3.3) | 56 (3.0) | 119 (3.4) | <0.001 |
| **1** | 978 (8.1) | 274 (4.0) | 65 (4.4) | 209 (3.9) |  | 334 (6.3) | 90 (4.9) | 244 (7.0) | <0.001 |
| **2** | 1473 (12.2) | 509 (7.5) | 106 (7.2) | 403 (7.6) |  | 511 (9.6) | 150 (8.1) | 361 (10.4) | <0.001 |
| **3** | 1689 (14) | 756 (11.2) | 162 (11.1) | 594 (11.2) |  | 631 (11.9) | 173 (9.3) | 458 (13.2) | <0.001 |
| **4+** | 7455 (61.7) | 5138 (75.9) | 1111 (75.9) | 4027 (75.9) |  | 3667 (69.0) | 1382 (74.7) | 2285 (65.9) | <0.001 |
| **Socioeconomic position, quintiles** |  |  |  |  |  |  |  |  |  |
| **5 (least deprived)** | 2374 (19.6) | 1181 (17.4) | 224 (15.3) | 957 (18.0) | 0.002 | 1151 (21.6) | 404 (21.8) | 747 (21.5) | 0.753 |
| **4** | 2539 (21) | 1260 (18.6) | 250 (17.1) | 1010 (19.0) |  | 1081 (20.3) | 389 (21.0) | 692 (20.0) | <0.001 |
| **3** | 2503 (20.7) | 1384 (20.4) | 297 (20.3) | 1087 (20.5) |  | 1119 (21.0) | 384 (20.7) | 735 (21.2) | <0.001 |
| **2** | 2341 (19.4) | 1531 (22.6) | 340 (23.2) | 1191 (22.4) |  | 1008 (19.0) | 355 (19.2) | 653 (18.8) | <0.001 |
| **1 (most deprived)** | 2332 (19.3) | 1415 (20.9) | 352 (24.1) | 1063 (20.0) |  | 959 (18.0) | 319 (17.2) | 640 (18.5) | <0.001 |

*Obtained from chi-squared or analysis of variance (ANOVA) as appropriate, test between men and women within each category of lives alone and lives with co-resident.

**Supplementary Table 4. Full model outputs for unadjusted, partially, and fully adjusted survival models.**

| **Outcome** | **Exposure** | **Model 1.**  **Unadjusted HR**  **(95%CI)** | **Model 2.**  **Partially adjusted HR**†  **(95%CI)** | **Model 3.**  **Fully adjusted HR**‡  **(95%CI)** |
| --- | --- | --- | --- | --- |
| **30-day emergency hospital admission** | Coresident: 0-1 long-term conditions & no dementia | *Reference* | *Reference* | *Reference* |
|  | Coresident: 2-3 long-term conditions & no dementia | 1.05 (0.85-1.30) | 1.02 (0.83-1.27) | 0.97 (0.78-1.20) |
|  | Coresident: 4+ long-term conditions & no dementia | 0.98 (0.80-1.19) | 0.92 (0.75-1.12) | 0.84 (0.69-1.03) |
|  | Coresident: dementia | 1.06 (0.67-1.69) | 0.97 (0.61-1.54) | 0.87 (0.54-1.38) |
|  | Lives alone | 1.10 (0.94-1.30) | 1.02 (0.86-1.20) | 0.96 (0.81-1.14) |
|  | Age (years) continuous | .. | 1.05 (0.97-1.13) | 1.03 (0.95-1.11) |
|  | Age (years) quadratic | .. | 1.00 (1.00-1.00) | 1.00 (1.00-1.00) |
|  | Sex: men | .. | *Reference* | *Reference* |
|  | Sex: women | .. | 0.73 (0.65-0.82) | 0.77 (0.69-0.87) |
|  | SEP quintile: 5 (least deprived) | .. | *Reference* | *Reference* |
|  | SEP quintile: 4 | .. | 0.97 (0.81-1.16) | 0.95 (0.79-1.14) |
|  | SEP quintile: 3 | .. | 1.09 (0.92-1.30) | 1.06 (0.89-1.26) |
|  | SEP quintile: 2 | .. | 1.17 (0.99-1.39) | 1.11 (0.93-1.31) |
|  | SEP quintile: 1 (most deprived) | .. | 1.26 (1.06-1.49) | 1.15 (0.97-1.37) |
|  | Number of long-term conditions (continuous) | .. | .. | 1.10 (1.09-1.12) |
| **One-year care home admission** | Coresident: 0-1 long-term conditions & no dementia | *Reference* | *Reference* | *Reference* |
|  | Coresident: 2-3 long-term conditions & no dementia | 1.42 (1.07-1.88) | 1.38 (1.04-1.83) | 1.32 (1.00-1.74) |
|  | Coresident: 4+ long-term conditions & no dementia | 1.47 (1.14-1.91) | 1.27 (0.98-1.65) | 1.20 (0.93-1.56) |
|  | Coresident: dementia | 3.52 (2.35-5.27) | 2.59 (1.73-3.88) | 2.38 (1.59-3.57) |
|  | Lives alone | 3.34 (2.69-4.15) | 2.33 (1.88-2.90) | 2.26 (1.81-2.81) |
|  | Age (years) continuous | .. | 1.23 (1.10-1.37) | 1.20 (1.08-1.35) |
|  | Age (years) quadratic | .. | 1.00 (1.00-1.00) | 1.00 (1.00-1.00) |
|  | Sex: men | .. | *Reference* | *Reference* |
|  | Sex: women | .. | 0.84 (0.75-0.9) | 0.87 (0.78-0.98) |
|  | SEP quintile: 5 (least deprived) | .. | *Reference* | *Reference* |
|  | SEP quintile: 4 | .. | 1.01 (0.85-1.19) | 1.00 (0.85-1.18) |
|  | SEP quintile: 3 | .. | 1.15 (0.98-1.35) | 1.13 (0.97-1.33) |
|  | SEP quintile: 2 | .. | 1.08 (0.92-1.27) | 1.05 (0.89-1.23) |
|  | SEP quintile: 1 (most deprived) | .. | 1.15 (0.97-1.35) | 1.08 (0.92-1.27) |
|  | Number of long-term conditions (continuous) | .. | .. | 1.08 (1.06-1.10) |
| **One-year mortality** | Coresident: 0-1 long-term conditions & no dementia | *Reference* | *Reference* | *Reference* |
|  | Coresident: 2-3 long-term conditions & no dementia | 1.18 (0.96-1.44) | 1.13 (0.93-1.38) | 1.06 (0.87-1.29) |
|  | Coresident: 4+ long-term conditions & no dementia | 1.24 (1.03-1.49) | 1.11 (0.92-1.33) | 0.99 (0.82-1.20) |
|  | Coresident: dementia | 1.57 (1.07-2.30) | 1.29 (0.88-1.88) | 1.11 (0.76-1.62) |
|  | Lives alone | 1.44 (1.23-1.68) | 1.19 (1.02-1.39) | 1.12 (0.95-1.31) |
|  | Age (years) continuous | .. | 1.00 (0.93-1.07) | 0.96 (0.90-1.04) |
|  | Age (years) quadratic | .. | 1.00 (1.00-1.00) | 1.00 (1.00-1.00) |
|  | Sex: men | .. | *Reference* | *Reference* |
|  | Sex: women | .. | 0.53 (0.48-0.59) | 0.57 (0.51-0.63) |
|  | SEP quintile: 5 (least deprived) | .. | *Reference* | *Reference* |
|  | SEP quintile: 4 | .. | 1.19 (1.01-1.39) | 1.17 (1.00-1.37) |
|  | SEP quintile: 3 | .. | 1.30 (1.12-1.52) | 1.26 (1.08-1.47) |
|  | SEP quintile: 2 | .. | 1.28 (1.10-1.50) | 1.20 (1.03-1.40) |
|  | SEP quintile: 1 (most deprived) | .. | 1.47 (1.26-1.71) | 1.32 (1.13-1.54) |
|  | Number of long-term conditions (continuous) | .. | .. | 1.13 (1.12-1.15) |

SEP = socioeconomic position

**Appendix 1. Prevalence of long-term conditions within hip fracture patients and co-residents.**

| **Long-term condition** | **Prevalence, N (%)** |
| --- | --- |
| Alcohol and substance misuse | 1148 (9.5) |
| Anaemia | 1223 (10.1) |
| Aneurysm | 359 (3) |
| Anxiety | 2199 (18.2) |
| Arrhythmia | 3847 (31.8) |
| Asthma | 1741 (14.4) |
| Autism | 10 (0.1) |
| Bipolar affective disorder | 176 (1.5) |
| Bronchiectasis | 364 (3) |
| Coronary artery disease | 4515 (37.3) |
| Cancer | 884 (7.3) |
| Cystic fibrosis | 10 (0.1) |
| Chromosomal abnormalities | 41 (0.3) |
| Chronic kidney disease | 6127 (50.7) |
| Chronic liver disease | 350 (2.9) |
| Chronic obstructive pulmonary disease | 2985 (24.7) |
| Chronic pancreatitis | 87 (0.7) |
| Connective tissue disorders | 1840 (15.2) |
| Dementia | 1747 (14.5) |
| Depression | 3601 (29.8) |
| Diabetes | 3310 (27.4) |
| Eating disorder | 40 (0.3) |
| Endometriosis | 101 (0.8) |
| Epilepsy | 14 (0.1) |
| Gout | 1634 (13.5) |
| Hearing impairment | 4212 (34.8) |
| Heart failure | 2263 (18.7) |
| Heart valve disease | 1902 (15.7) |
| Hypertension | 11388 (94.2) |
| Inflammatory bowel disease | 365 (3) |
| Meniere's disease | 503 (4.2) |
| Multiple sclerosis | 78 (0.6) |
| Osteoarthritis | 7553 (62.5) |
| Osteoporosis | 5712 (47.2) |
| Peripheral arterial disease | 1440 (11.9) |
| Paralysis | 35 (0.3) |
| Parkinson's disease | 496 (4.1) |
| Peptic ulcer | 1336 (11.1) |
| Peripheral neuropathy | 1013 (8.4) |
| Post-traumatic stress disorder | 43 (0.4) |
| Schizophrenia | 231 (1.9) |
| Stroke and transient ischaemic attack | 2789 (23.1) |
| Thyroid disorders | 2640 (21.8) |
| Tuberculosis | 310 (2.6) |
| Visual impairment | 884 (7.3) |
| Venous thromboembolism | 823 (6.8) |
